# Seasonality and Locality Affect the Diversity of *Anopheles gambiae* and *Anopheles coluzzii* Midgut Microbiota from Ghana

**DOI:** 10.1371/journal.pone.0157529

**Published:** 2016-06-20

**Authors:** Jewelna Akorli, Mathilde Gendrin, Nana Adjoa P. Pels, Dorothy Yeboah-Manu, George K. Christophides, Michael D. Wilson

**Affiliations:** 1 Department of Parasitology, Noguchi Memorial Institute for Medical Research, College of Health Sciences, University of Ghana, P.O. Box LG 581, Legon, Accra, Ghana; 2 Department of Bacteriology, Noguchi Memorial Institute for Medical Research, College of Health Sciences, University of Ghana, P.O. Box LG 581, Legon, Accra, Ghana; 3 Department of Life Sciences, Imperial College London, Sir Alexander Fleming Building, South Kensington Campus, Imperial College Road, SW7 2AZ London, United Kingdom; Centro de Pesquisas René Rachou, BRAZIL

## Abstract

Symbiotic bacteria can have important implications in the development and competence of disease vectors. In *Anopheles* mosquitoes, the composition of the midgut microbiota is largely influenced by the larval breeding site, but the exact factors shaping this composition are currently unknown. Here, we examined whether the proximity to urban areas and seasons have an impact on the midgut microbial community of the two major malaria vectors in Africa, *An*. *coluzzii* and *An*. *gambiae*. Larvae and pupae were collected from selected habitats in two districts of Ghana during the dry and rainy season periods. The midgut microbiota of adults that emerged from these collections was determined by 454-pyrosequencing of the *16S* ribosomal DNA. We show that in both mosquito species, *Shewanellaceae* constituted on average of 54% and 73% of the midgut microbiota from each site in the dry and rainy season, respectively. *Enterobacteriaceae* was found in comparatively low abundance below 1% in 22/30 samples in the dry season, and in 25/38 samples in the rainy season. Our data indicate that seasonality and locality significantly affect both the diversity of microbiota and the relative abundance of bacterial families with a positive impact of dry season and peri-urban settings.

## Introduction

Mosquito midgut microbiota has become an interesting field in mosquito vector biology, as it has been shown to form an integral part of the mosquito life history [1]. A comparison of septic and aseptic (antibiotic-treated) mosquitoes has portrayed the importance of symbiotic bacteria in the development [2,3], physiology [4] and immunity [5] of the mosquito host. Natural midgut microbiota induce antimicrobial genes and decrease the mosquito’s susceptibility to *Plasmodium* [5,6].

The *Anopheles gambiae* complex comprises of major malaria vectors, widespread in sub-Saharan Africa [7]. Among these, *Anopheles gambiae* (formerly ‘S’ form of *An*. *gambiae* Giles) and *An*. *coluzzii* Coetzee & Wilkerson *sp*.*n*. (formerly ‘M’ form of *An*. *gambiae* Giles) are two sibling species which are principal vectors of malaria [8], and occur in sympatry in most parts of West Africa [9]. Although morphologically indistinguishable, they show certain molecular, physiological, and ecological differences [10,11]. *Anopheles gambiae* exhibits a significantly higher permissiveness to *P*. *falciparum* and more insecticide resistance than *An*. *coluzzii* [12–15].

The midgut microbiota of *Anopheles* influences the mosquito vector competence to *Plasmodium* [5,6,16] by inducing immune responses [6], and having direct anti-parasitic activity [16]. It also affects the mosquito vectorial capacity by affecting the mosquito fitness [17,18]. Its composition is highly variable between individual field mosquitoes [19], which may partly explain the variability in mosquito infection levels in the field [20]. Although the mosquito microbiota composition is largely influenced by their aquatic breeding environment [3,21,22], the exact factors defining the structure of the adult mosquito microbiota are currently unknown [23]. Identifying these factors and widely characterising the native microbiota composition of mosquito disease vectors will however be essential for bacteria-mediated disease control mechanisms to be useful and feasible. In this study, we investigated the midgut microbiota of female adult *An*. *gambiae* and *coluzzii* that emerged from larvae and pupae collected from breeding sites in two districts in Ghana; urban Accra Metropolis and villages in Kintampo North Municipality. The midgut microbiota composition and richness were compared between sites and between the two mosquito species.

## Materials and Methods

### Study sites

**Fig 1:**
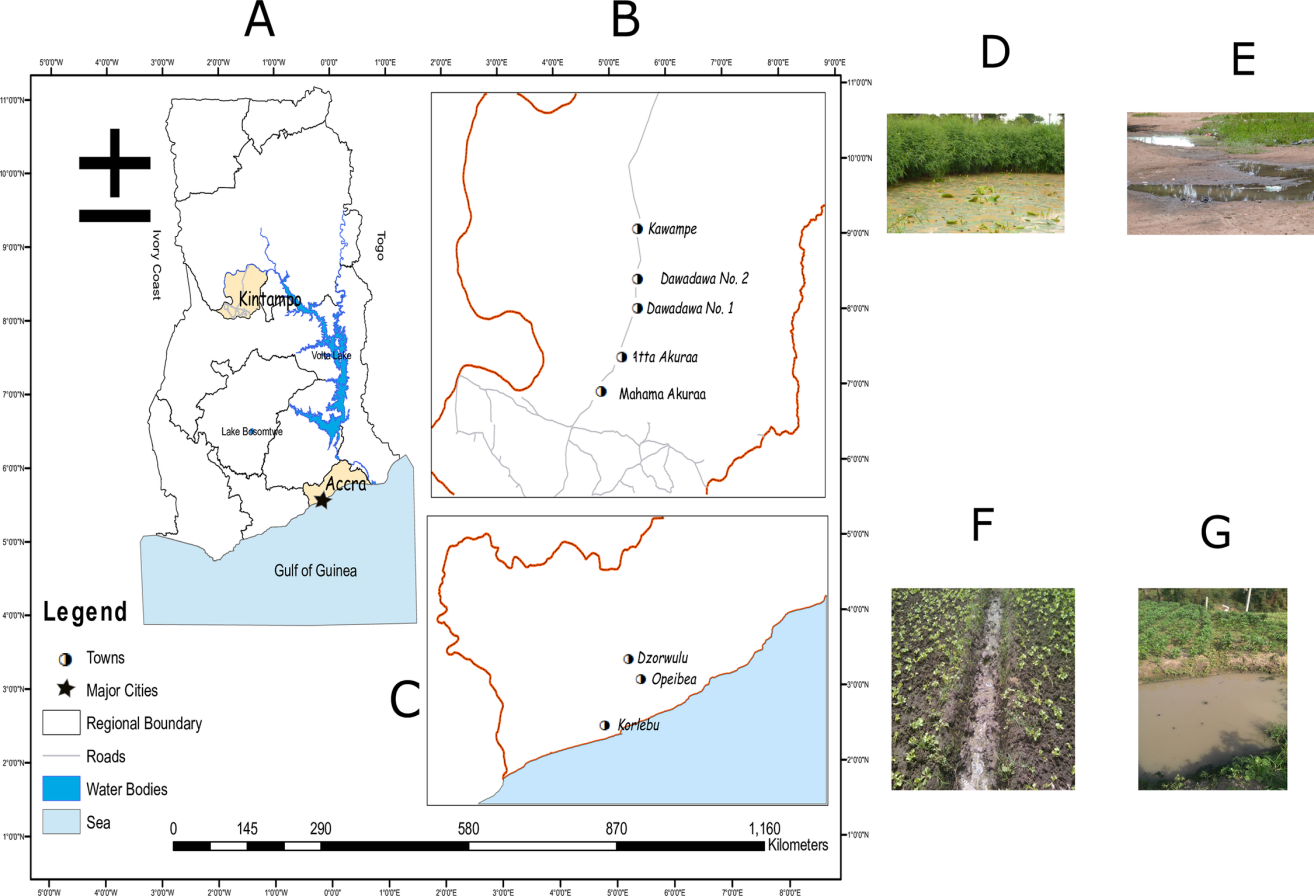
Map of Ghana showing the studied districts and examples of habitats sampled.

Mosquito breeding sites in two selected districts, Accra Metropolis and Kintampo North Municipality, were sampled (Fig 1A, S1 Table). Accra Metropolis, one of the 10 administrative districts in the Greater Accra Region of Ghana, is largely urban. It incorporates the capital, Accra, and inhabits approximately 1.8 million people; i.e. 7.5% of the country’s population [24]. The Greater Accra Region forms part of the coastal savannah ecological zone of the country. About 430km north of the Greater Accra Region is the Brong-Ahafo Region, with Kintampo North Municipality as one of its 22 districts. With a population of about 95,000 [24], Kintampo North is mostly rural with villages located along the stretch of the main town of Kintampo towards the north. The ecological zone here is described as the forest-savannah transitional zone.

Breeding sites close to six villages in the Kintampo North Municipality were sampled; Atta Akuraa (Ki_ATT), Dawadawa No1 (Ki_DD1), Dawadawa No2 (Ki_DD2), Kawampe (Ki_KAW), Mahama Akuraa (Ki_MAH) and Tahiru Akuraa (Ki_TAH) (Fig 1B). In Accra Metropolis, three breeding sites were sampled for the study; Korle-Bu (Ac_KBU), Dzorwulu (Ac_DZW) and Opeibea (Ac_OPB) (Fig 1C). All the sampling sites in Kintampo North were non-cultivated but, had adequate vegetation cover (Fig 1D). In the rainy season, some samples were obtained from water that usually collected in puddles after a rainfall (Fig 1E). Breeding sites in Accra Metropolis were irrigation sites for cultivation of vegetables (Fig 1F and 1G). Samples were either obtained from water that had collected between the raised beds (Fig 1F), and/or from dug-out wells which collected rain water for watering vegetables (Fig 1G).

### Mosquito collection and dissections

Where required, permission to sample breeding habitats was sought from community leaders and farmland owners. Pupae and 3^rd^-4^th^ instar larvae of *Anopheles* mosquitoes were collected from mosquito breeding habitats between February-March (dry season) and July-August (rainy season) in 2014. Mosquito samples were brought to the laboratory in water samples from their respective collection sites. Upon arrival at the laboratory, pupae were transferred into plastic cups along with their breeding water sample and, placed in labelled cages that had been wiped with 70% ethanol. The remaining larvae were carefully poured into larval trays, and maintained in their water samples without feeding them. The larval trays were inspected every day, and pupae were collected and placed in cages. Larvae were discarded 5 days post field collections. Breeding sites in Accra only needed to be visited once to obtain appreciable number of female mosquitoes for the dissections, while some sample sites in Kintampo North Municipality were visited twice in a week.

One-day old, unfed female adults were morphologically identified using identification keys [7], and those identified as members of *An*. *gambiae* complex were preserved at -20°C. Mosquitoes were ‘surface-sterilized’ in 0.5% (v/v) bleach for 10 mins, and 1 min each in 1X PBS, 70% ethanol, and finally 1X PBS, before dissection. Dissecting pins, forceps and microscope slides were cleaned with 0.5% (v/v) bleach and 70% ethanol. Each mosquito was dissected in a drop of filter-sterilized 1X PBS, on a microscope slide. Midguts were preserved singly in 1.5ml microcentrifuge tubes containing absolute ethanol. The head and thorax of each dissected mosquito was also preserved in absolute ethanol in a separate tube. The tips of dissecting pins and forceps were cleaned with bleach and ethanol between each dissection to avoid contamination between sample midguts. Mock dissections were performed by repeating the same dissection procedure but without placing a mosquito on the microscope slide.

### DNA extraction for *Anopheles* species identification

Genomic DNA was extracted from individual mosquito carcasses (head and thorax) using a protocol adapted from [25] (Protocol 2). Briefly, each sample was homogenized in 200μL of Lysis buffer (0.1M Tris (pH 8.0), 0.05M EDTA, 0.5M NaCl, 1.3% SDS and 50μg/ml RNase A), and 4μL proteinase K (Qiagen) was added. The homogenate was mixed by vortexing and incubated at 65°C for 30mins. Sixty microliters (60μL) of 5M potassium acetate was added, mixed by inverting tube, and incubated on ice for 30mins. Each supernatant was transferred to a new tube after centrifuging homogenates at 10,000 rpm for 10mins. DNA was precipitated with 500μL of ice-cold absolute ethanol and incubated at -20°C for 10mins. Samples were centrifuged at 13,000rpm for 5mins, ethanol discarded, and DNA pellet washed with 200μL of 70% ethanol. DNA pellets were re-suspended in AE buffer (Qiagen). Identification of *An*. *gambiae* and *An*. *coluzzii* was performed using primers and protocol described by [26].

### Preparation of mosquito midguts for pyrosequencing

After species identification, mosquito midgut samples were pooled in groups of 2–5 (average number of midguts per pool = 3.10) based on their species, collection site and sampling season. Only one tube had a single midgut. Total DNA was extracted from each pool using the QiAamp DNA micro kit (Qiagen), following manufacturer’s manual. All steps involving opening of the tubes were performed under an airflow chamber. DNA was finally eluted in 100μL of AE buffer (Qiagen). The Qubit Fluorometer 2.0 (Invitrogen) was used to quantify 10μL of mock extract. No DNA was detected in the mock samples.

DNA extracted from midgut samples were prepared for pyrosequencing by amplifying the bacterial 16S rDNA and tagging each sample pool with 454 FLX Titanium Multiplex Identifiers (MIDs) (Roche). The mock, although it showed no detectable DNA during quantification, was taken through the rest of the processes to check for contamination in downstream analyses. In a first reaction (Nest I), ~1500bp of the bacterial 16SrDNA was amplified using universal bacterial primers 27F (5´ GAG TTT GAT CMT GGC TCAG 3´) and slightly modified 1525R- (5´ GAA GGA GGT GAT CCA NCC 3´). Each reaction tube contained 4.1μL UV-irradiated 18.2mQ water, 5μL of 2x Phusion High Fidelity Master Mix (New England Biolabs, MA, USA) Master Mix, 0.2μL each of 20μM forward and reverse primer and 0.5μL DNA template. Cycling conditions included initial denaturation at 98°C for 3mins, 35 cycles of denaturation at 98°C for 30s, annealing at 55°C for 10s and extension for 72°C for 20s. Final extension was performed at 72°C for 7mins, and samples were kept at 4°C. The presence of bacteria 16S rDNA was confirmed by loading 3μL of PCR product on a 2% ethidium bromide stained agarose gel, and visualizing on a UV transilluminator (Cleaver Scientific).

The PCR product from Nest I reaction was used in a second PCR to amplify ~345bp of the hypervariable V3 region of the bacterial 16S rDNA [27] with the primers 338–358 F (5′ ACT CCT ACG GGA GGC AGC AGT 3′) and 683–700 R (5′ CGM ATT TCA CCK CTA CAC 3′) [28], designed according to 454 GS-FLX Titanium primer recommendations (Roche). Each sample was given a unique set of barcoded forward and reverse primers (S2 Table). A total volume of 40μL reaction mixture was prepared for each sample; 15.2μL UV-irradiated 18.2mQ water, 20μL 2x Phusion High Fidelity Master Mix, 0.8μL of 20μM 454 primer mix, and 4μL Nest 1 PCR product. The PCR for each sample was performed in 4 x 10μL aliquots of the reaction mix. Cycling conditions were similar to those of Nest I, except for the annealing temperature which was 59°C. After the PCR, the 4 x 10μL aliquots for each sample were pooled, and 3μL were visualized on a 2% agarose gel stained with ethidium bromide. For each seasonal collection, equimolar concentrations of amplicons were pooled after quantifying with the Qubit Fluorometer 2.0 (Invitrogen). Pooled samples were purified by gel extraction using the MinElute Gel Extraction kit (Qiagen), following manufacturer’s recommendations. Each pool of library (one for each season) was sequenced on a Junior^+^ System Genome Sequencer at the Department of Biochemistry, University of Cambridge, UK.

### Processing of sequence data

Sequences for dry and rainy season collections were analysed separately, but with similar pipeline criteria. Our sequencing depth produced 98,303 and 92,788 sequences for dry and rainy season, respectively. The dry season pool had an average read length of 379.69 (standard deviation = 24.15), while the rainy season pool recorded an average read length of 380.07 (standard deviation = 24.13). Sequences from both pools had a median and modal length of 383.0. All sequences were filtered using Geneious vR7.1.2 [29] to include only those with length 340-400bp, and passed our primer MIDs combinations without a nucleotide mismatch.

The filtered sequences were taken through the QIIME (Quantitative Insights Into Microbial Ecology) software [30] for chimera detection, taxonomic assignments and rarefaction. Operational Taxonomic Units (OTUs) were defined by sequences with at least 97% nucleotide similarity by the furthest neighbour method [31]. OTUs were aligned to the Greengenes core reference [32] using the QIIME default Python Nearest Alignment Space Termination (PyNAST) [33] and uclust pairwise alignment methods [34]. The sequences were screened for identification and removal of chimeras with the ChimeraSlayer algorithm [35]. To be able to identify the bacterial sequences, OTUs were taxonomically classified to the species level with the RDP Classifier II [36] based on the SILVA119 ribosomal database [37,38] using the algorithm’s default minimum confidence level of 0.5.

To account for what may be potential contamination in our test sequences, the mock and test samples were processed similarly through taxonomy. As contamination is mostly expected to influence the results of low abundance samples, a linear regression and Spearman’s rho test (ρ) between amplicon concentration and relative bacterial abundance would give a significantly negative correlation [39,40]. This analysis indicated that *Halomonas*, which represented 83.7% of bacteria in the mock, was a laboratory contamination. The sequences for these were removed from the test samples for downstream analyses (more details in S1 Methods, S1 and S2 Figs). Rarefaction curves were used to assess the sequencing depth (S3 Fig).

### Alpha and beta diversity analyses

Diversity analyses and ordination methods were performed in the VEGAN package [41] for community ecological analyses on the R statistical platform [42]. The bacterial abundance data matrices were first transformed by the Hellinger transformation [43]. Alpha diversity for each habitat was then estimated with Shannon-Weaver index on the transformed community matrices of OTUs and families, and tested for significant differences between groups with non-parametric Kruskal-Wallis test.

We determined how variables such as collection sites, seasons and mosquito species correlated with bacterial family abundance by performing Canonical Correspondence Analyses (CCA) on a combined matrix, obtained by pooling data sets from both seasons, and transformed by chi-square transformation [44]. For the CCA triplot, midgut samples and collection sites were scaled proportional to the Eigenvalues (Scaling Type I). Dissimilarity indices were built based on Bray-Curtis [45], and test of significance for the dissimilarity index matrices was tested with ANOSIM [42]. The significance of each explanatory variable on the bacteria family distribution was determined with ADONIS [42]. Further analyses to detect bacteria that contributed to observed variation within mosquito species were performed at the bacteria genus level. Statistical analyses were conducted using non-parametric Kruskal-Wallis and Mann-Whitney tests.

## Results

### Distribution of *Anopheles gambiae* and *Anopheles coluzzii*

Mosquitoes were collected from breeding sites situated in rural and urban areas in Ghana. Prior to analysing the mosquito microbiota, we first characterized the mosquito species composition in each habitat and each season. A total of 212 *An*. *gambiae* (dry season = 137; rainy season = 75) were raised to adults in the laboratory (Table 1). In the dry season, the proportion of *An*. *coluzzii* (57%) and *An*. *gambiae* (43%) were not significantly different (Mann-Whitney U = 14.5; *p* = 0.60). The distribution of the two mosquito species between rural and urban habitats was also similar (Kruskal test: *An*. *coluzzii*- *p* = 0.05; *An*. *gambiae*- *p* = 0.35). In the rainy season, the collection contained predominantly *An*. *gambiae* (88%) (Mann-Whitney U = 6.5; *p* = 0.004) and *An*. *coluzzii* were only sampled from 2/8 sites (Table 1). In both periods, the distribution of mosquito species varied among breeding sites (Chi-square: dry season- *p*<0.0001; rainy season- *p*<0.0001).

**Table 1:** Distribution of mosquitoes dissected over two collection periods; dry and rainy seasons. Numbers in brackets are the number of midgut pools.

		Dry season			Rainy season		
District	Collection site	*An*. *coluzzii*	*An*. *gambiae*	TOTAL	*An*. *coluzzii*	*An*. *gambiae*	TOTAL
Accra Metropolis	Ac_DZW	7 (2)	9 (2)	16	0	10 (5)	10
	Ac_OPB	2 (1)	20 (4)	22	0	10 (5)	10
	Ac_KBU				8 (4)	0	8
Kintampo North Municipality	Ki_ATT	9 (2)	9 (2)	13	0	8 (4)	8
	Ki_KAW	20 (4)	11 (3)	31	0	10 (5)	10
	Ki_DD1	20 (4)	5 (1)	30	-	-	-
	Ki_DD2	20 (4)	5 (1)	25	-	-	-
	Ki_DD1/2	-	-		0	10 (5)	10
	Ki_TAH	-	-		1 (1)	6 (3)	7
	Ki_MAH				0	12 (6)	12
**TOTAL**	** **	**78**	**59**	**137**	**9**	**66**	**75**

### Dry season and urban locality positively affect mosquito microbiota diversity

**Fig 2:**
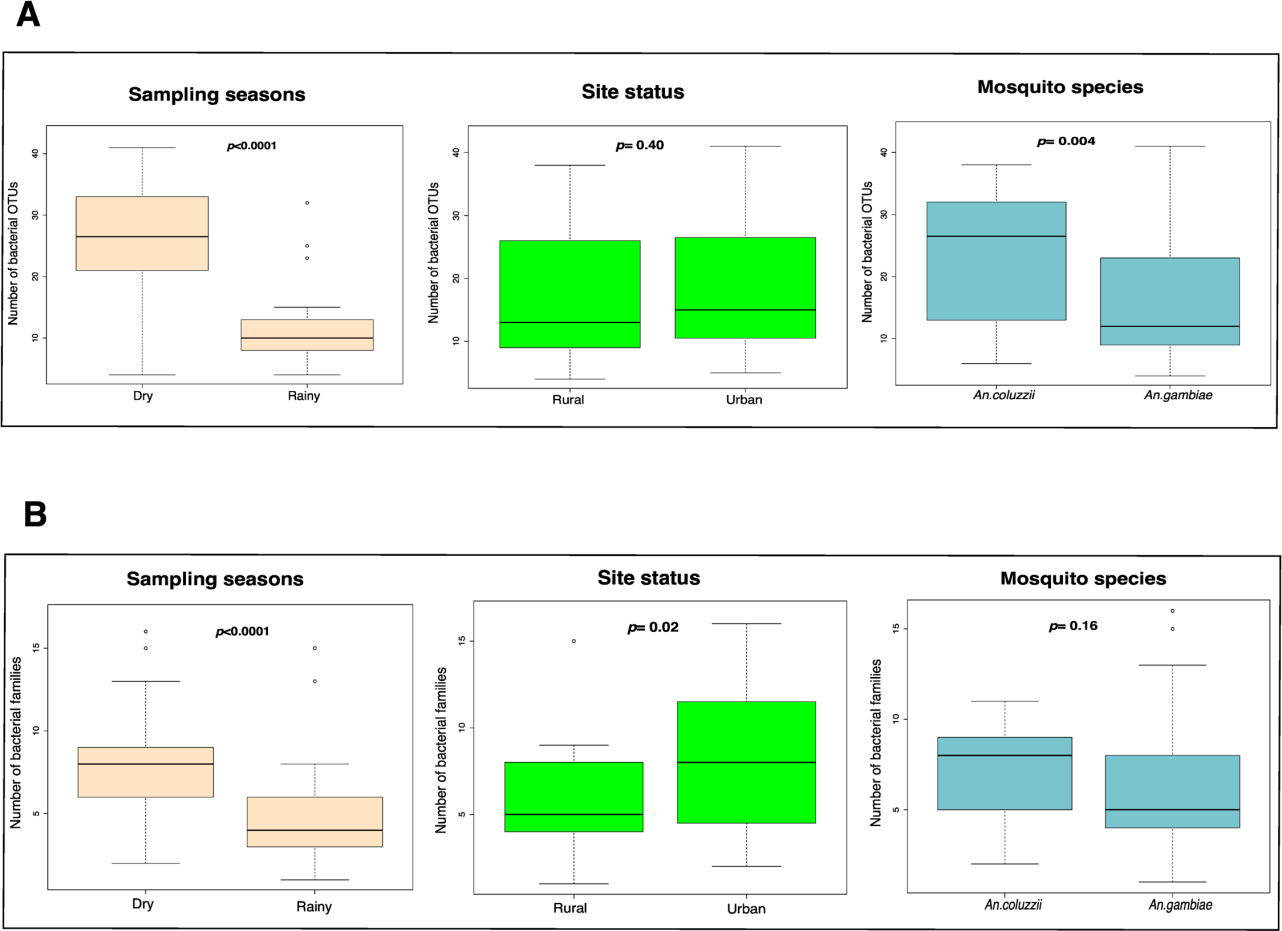
**Boxplot comparing OTU (A) and bacterial family (B) abundances for mosquito samples.** Plots show distribution of bacteria between mosquito samples categorized under different sampling season, site status, and mosquito species. Black lines indicate medians.

We analysed the microbiota composition in the midgut of adult mosquitoes by 454-pyrosequencing on the 16S rDNA gene in pools of 2–5 mosquitoes. A total of 144 and 79 OTUs were identified for the dry and rainy season data, respectively (S3 Table). Although these indicate high OTU richness, especially in the dry season, this was not the case at the sample level, where only 27 (23–29; CI = 95%) and 11 (9–13; CI = 95%) OTUs/sample were observed on average in the dry and rainy season, respectively (Fig 2 and S3 Table). As the number of guts was not the same in all samples, we normalized the data (see S1 Methods) taking into account the number of guts in each sample and the shared proportion between individuals found in our previous study [19]. The results found after normalization were similar to the initial results (S4 Fig) ruling out the possibility that the differences in diversity were due to differences in the number of guts in the sample.

**Fig 3:**
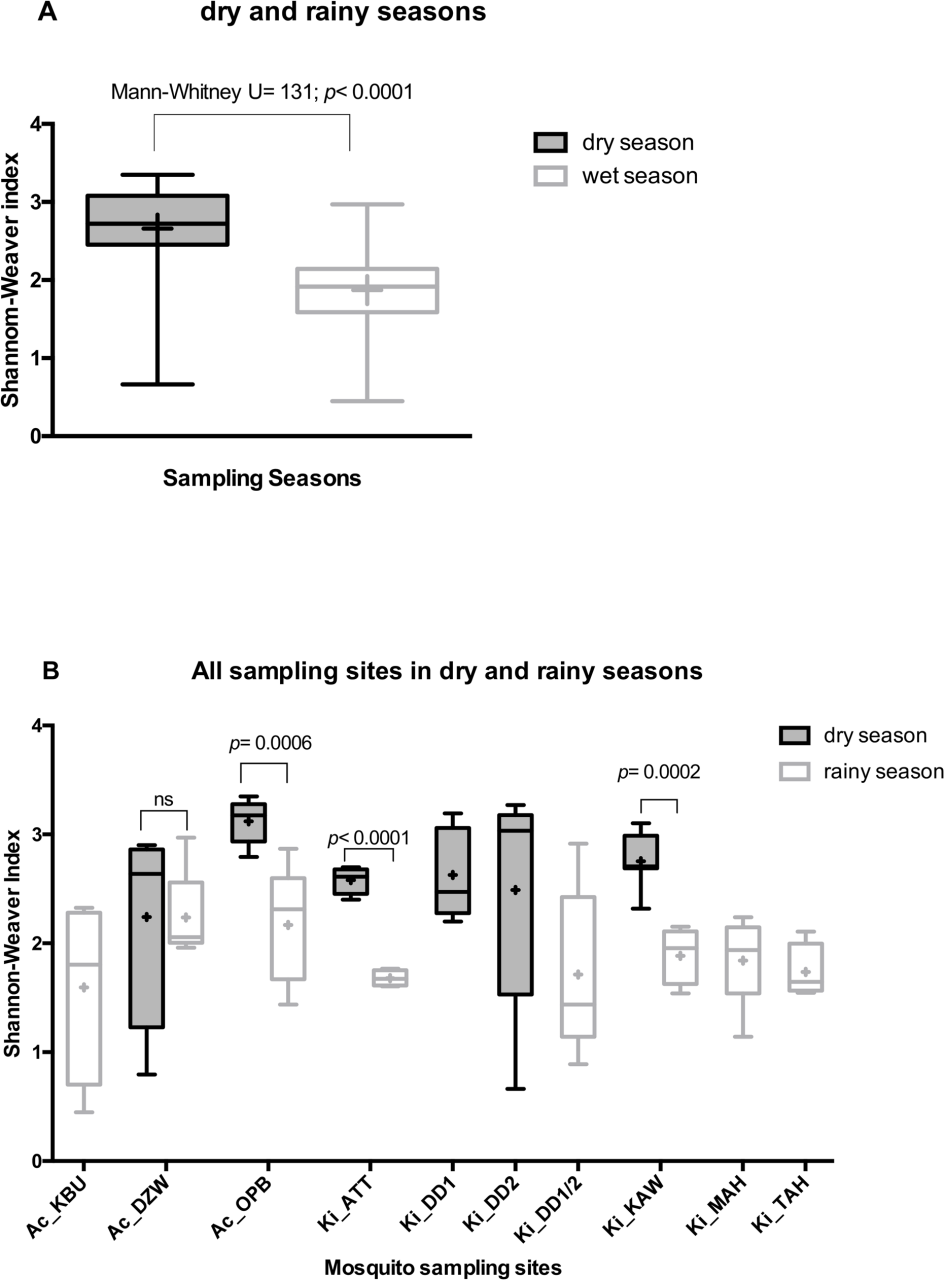
Shannon-Weaver indices compared among samples collected in dry and rainy season. Crosses in the box indicate the mean, and black lines are medians.

The Shannon-Weaver indices further emphasised the low diversity of bacteria OTUs per sample from each location (maximum average = 3.1, minimum average = 1.6) (Fig 3). The average diversity for the dry season was, however, significantly higher than the rainy season (Shannon index: dry season = 2.7 (2.4–2.9; CI = 95%), rainy season = 1.9 (1.7–2.0; CI = 95%), *p*<0.0001) (Fig 3A). The same seasonal differences in species richness were observed when only testing sites that were sampled in both seasons (Mann-Whitney U = 45, *p*<0.0001) (Fig 3B). A higher diversity was also observed at the bacterial family level, where the average number of bacterial families observed was 6.1 in the dry season and 4.9 in the rainy season (Fig 2B). Aside the season, we observed that the urban locality of larvae sampling sites also had a positive effect on the microbiota diversity at the family level (Fig 2B), and that *An*. *coluzzii* harboured significantly more diverse microbiota than *An*. *gambiae* when considering the number of OTUs (Fig 2A).

### Dominance of *Shewanellaceae* during both seasons

**Fig 4:**
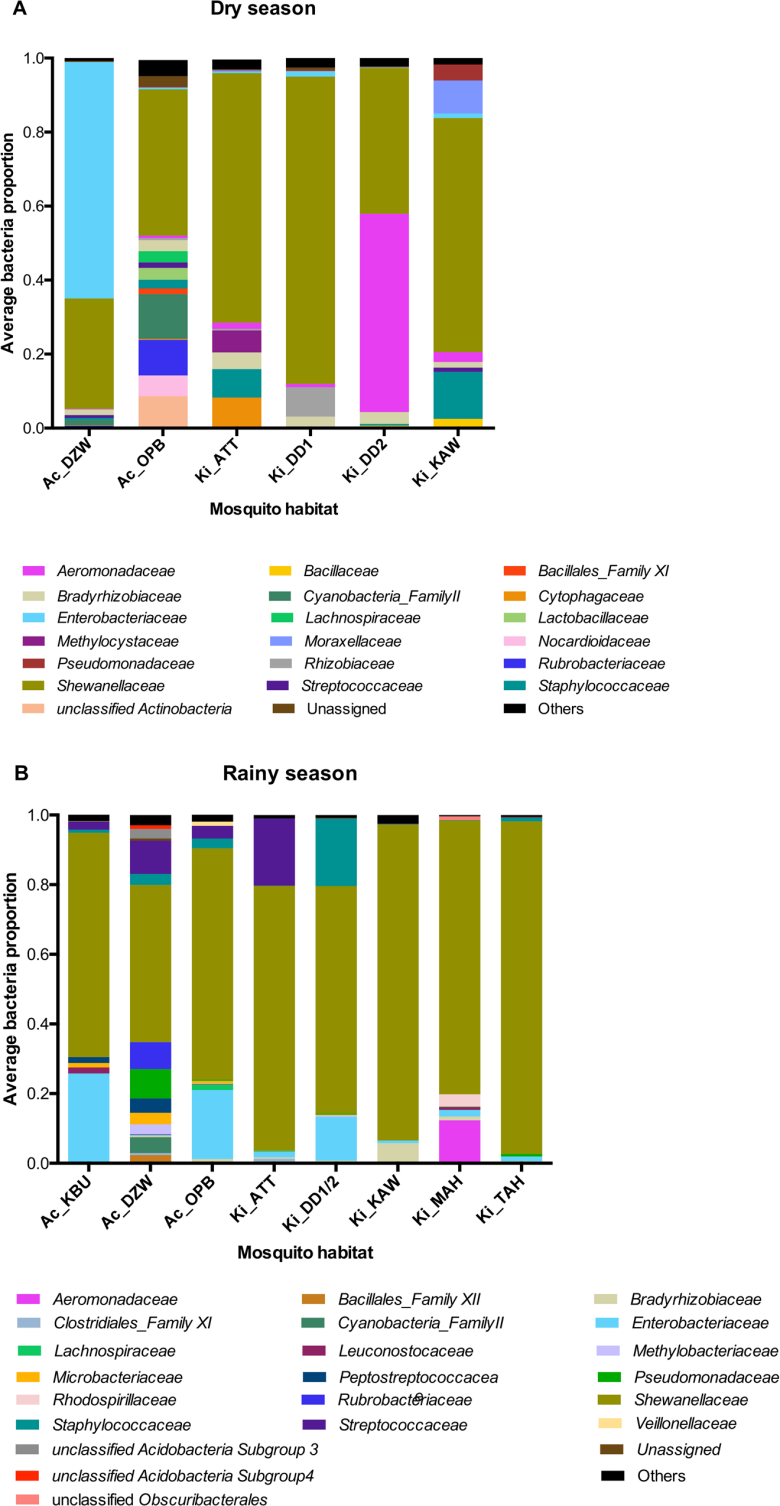
**Bacterial family proportions in mosquito midguts sampled from breeding sites in Accra Metropolis (Ac_KBU, Ac_DZW, Ac_OPB) and Kintampo North Municipality (Ki_ATT, Ki_DD1, Ki_DD2, Ki_DD1/2, Ki_KAW, Ki_MAH, Ki_TAH) during the dry (A) and rainy (B) seasons.** Families representing ≥0.01 proportions in each site are shown; all families <0.01 are grouped as ‘Others’.

We analysed the microbiota composition at the family level and observed that *Shewanellaceae* family represented on average 54% of the mosquito microbiota in the dry season and 73% in the rainy season (Fig 4), but this seasonal increase was not significant (Mann-Whitney U = 11 *p* = 0.1). *Shewanellaceae* was similar between *An*. *coluzzii* and *An*. *gambiae* (Average = 64%, Mann-Whitney U = 462, *p* = 0.48). Rural habitats, however, had a significantly higher proportion of *Shewanellaceae* (72%) than urban habitats (49%) (Mann-Whitney U = 291, *p* = 0.003). Bacterial families *Enterobacteriaceae* and *Aeromonadaceae*, commonly associated with *Anopheles* mosquitoes [1,19], were only found above 1% in 21/68 samples and 11/68 samples, respectively. When present, their contribution was however substantial, as *Enterobacteriaceae* represented up to 99.8% and *Aeromonadaceae* 98% of the total microbiota in some samples (S4 Table).

### Factors contributing to the beta-diversity

**Fig 5:**
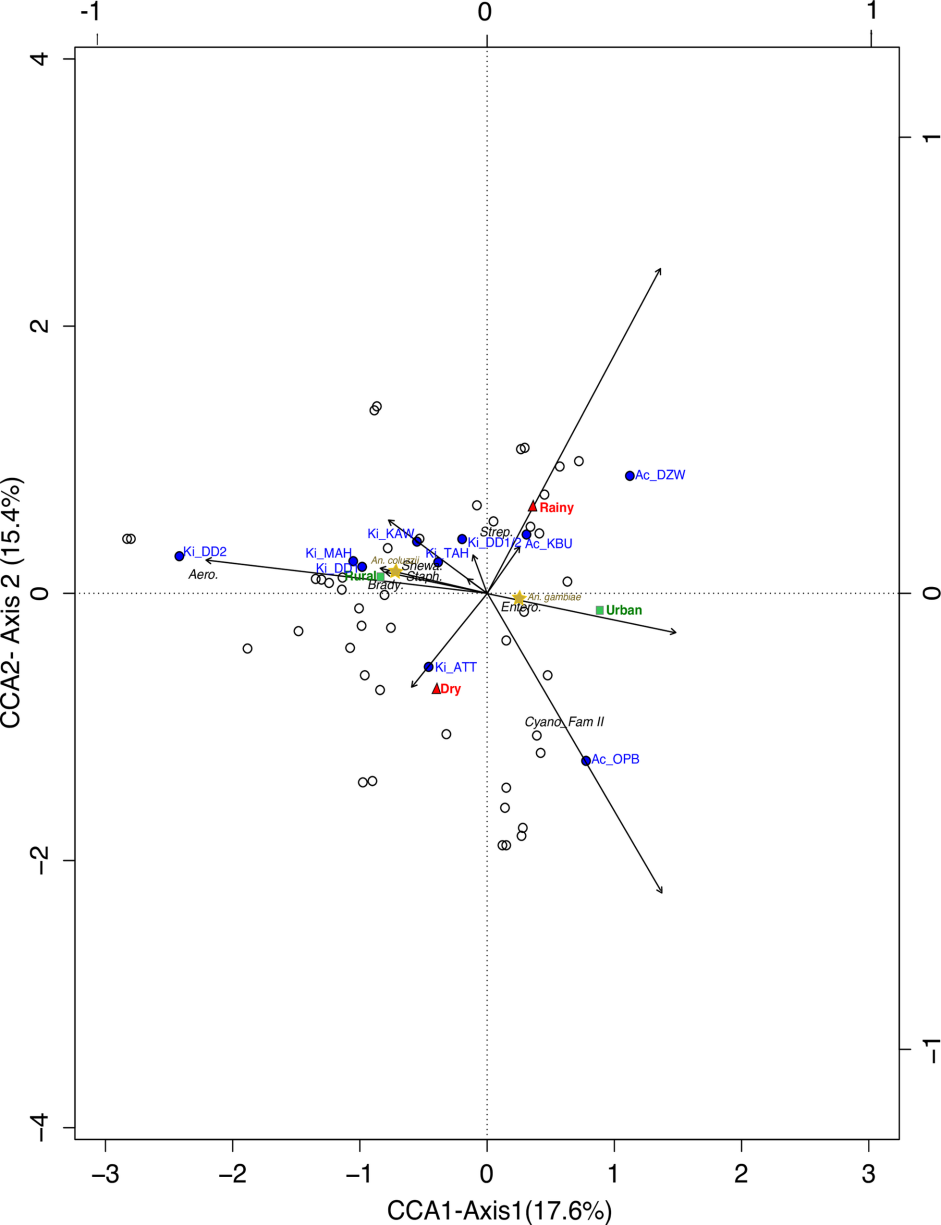
Canonical Correspondence Analysis (CCA) for microbiota composition between breeding sites in the dry and rainy season. Filled circles represent breeding sites and hollow circles represent samples (taxonomic level = family). Bacterial families with ≥0.01 relative abundance are indicated by name. Filled squares, triangles and stars represent site status (rural, urban), sampling season (dry, rainy), and mosquito species respectively. The length of each arrow indicates the strength of the variable that explains the bacteria dispersion observed. Bottom x-axis and left y-axis are scales for factors plotted, while top x-axis and right y-axis are scales for the biplot (arrows).

Besides species diversity, we assessed which factors play a role in the microbiota composition. Based on the bacterial family distribution, we observed that seasons (ANOSIM: R = 0.33, *p* = 0.001) and urban vs rural localities (ANOSIM: R = 0.39, *p* = 0.001) had a significant impact on the microbiota composition, while the effect of species was only marginally significant (ANOSIM: R = 0.08, *p* = 0.067). To go further, we performed a CCA including all our variables, i.e. location, collection season, mosquito species and status of collection site (rural or urban) (Fig 5). The total inertia in the matrix data was 13.8, and the constrained inertia was 3.4 therefore, the constraining variables could only explain 25% of the family distribution, leaving a large percentage unexplained. When considering each variable separately, location, collection site status, season and mosquito species constrained 20.3%, 4.0%, 3.2% and 2.1% of the inertia, respectively. Interestingly, the rural and *An*. *coluzzii* states almost overlap on the CCA chart (Fig 5), suggesting that a typical microbiota composition of an *An*. *coluzzii* mosquito would be similar to a typical composition in a rural mosquito.

The first two constrained axes had the highest Eigenvalues (Axis 1 = 0.60, Axis 2 = 0.53), but, their contributions to the constrained inertia was altogether less than 50% (Axis 1 = 18%, Axis 2 = 15%) (Fig 5). Permutational test (R: ADONIS function (n = 999 permutations)) indicated that breeding sites (*F*_(9, 56)_ = 1.74, *p* = 0.002), seasons (*F*_(1, 56)_ = 2.46, *p* = 0.012) and locality (*F*_(1, 56)_ = 5.36, *p* = 0.001), but not mosquito species (*F*_(1, 56)_ = 1.0, *p* = 0.42), have a significant effect on the distribution of bacteria families. These results, together with the observed effects on bacterial family richness (Fig 2), suggest that seasons and locality affect the microbiota composition while mosquito species do not. Although most of the bacteria were ‘rare’, low in abundance and randomly distributed, some bacteria families (with relative abundance ≥0.01) showed relatedness to certain sites as indicated by the directions of the arrows towards the position of the sites on the CCA plot (Fig 5); *Aeromonadaceae* with Ki_DD2, *Cyanobacteria_Family II* with Ac_OPB, were both significantly higher in abundance than realised in other breeding sites (S5 Table).

The mosquito microbiota is known to be relatively simple within an individual but highly variable between individuals [19]. We investigated the source of variation by analysing dissimilarity matrices between samples in the dry and rainy seasons. Bacterial family composition significantly differed between *An*. *gambiae* samples from the dry (ANOSIM: R = 0.11, *p* = 0.04), but not from the rainy season (ANOSIM: R = 0.04, *p* = 0.35). No significant differences were found among *An*. *coluzzii* samples. To further investigate the source of this difference observed in the dry season, the bacteria genera were analysed, first between mosquito species (Mann-Whitney U = 76, *p* = 0.13), and then within mosquito species. When comparing the relative contribution of the main genera (>1%), microbiota composition differed significantly among *An*. *gambiae* samples (Kruskal-Wallis test: *p* = 0.006), but not among *An*. *coluzzii* samples (Kruskal-Wallis test: *p* = 0.97). Among *An*. *gambiae* samples, the difference was observed between samples of different breeding sites but samples from the same collection site did not differ in the composition of their microbiota. *Aeromonas*, *Shewanella* and *Thorsellia* were the bacteria genera found to be significantly varying in abundance between breeding sites (S6 Table). No significant differences were observed between *An*. *gambiae* samples of the dry and of the rainy season (Mann-Whitney U = 90, *p* = 0.06).

## Discussion

*An*. *gambiae* has been known to thrive in temporary puddles of water collected after rains, and are mostly found in rural settings. However, urbanization of this species of mosquito and its adaptation to permanent standing water has recently become a concern (reviewed in [46]). The environmental conditions in which mosquito larvae develop are key determinants within the vector [3,21], and this could have important implications on the vector competence of the mosquitoes (reviewed in [47]). In this study, we compared the midgut microbiota of *An*. *gambiae* and *An*. *coluzzii* sampled from mosquito breeding sites in urban and rural settings during the dry and rainy season with the aim of identifying differences in mosquito midgut microbiota. Generally, our data suggest that the dry season, urban settings and *An*. *coluzzii* species have a positive effect on the microbiota diversity. *An*. *gambiae* and *An*. *coluzzii* did not significantly differ in the overall composition of their midgut microbiota, but differences were observed among *An*. *gambiae* coming from different collection sites, especially during the dry season. This corroborates previous data showing the larval breeding site has a significant impact on the adult mosquito midgut microbiota composition [22].

We investigated the natural midgut microbiota of one-day old female mosquitoes. Field-caught adult mosquitoes are often studied for their midgut microbiota, but as sugar and blood meals have different effects on the microbiota, the differences between individuals could be difficult to explain as previous feeding history is unknown [19]. The collection of pupae and late instar larvae ensured that a good representation of the midgut microbiota obtained from the breeding sites by the emerging adult is captured. Using one-day old, unfed mosquitoes also allowed the comparison of mosquitoes with known feeding histories and of the same age. Although this does not represent the bacteria at the time of parasite exposure because the mosquitoes were not blood fed, the microbiota identified shows the diversity of bacteria species obtained from the larval environment. Such information could be useful in identifying bacteria species that can be used in ‘symbiotic control’ strategies for disease control [48], as diversity of bacteria differs significantly between larval breeding water and mosquito [49].

The microbiota catalogue of the *Anopheles* mosquitoes collected in this study was remarkably different midgut microbial composition from what has been reported in one-day old, unfed *An*. *gambiae* from Kenya [1]. The most striking difference is that bacterial family *Shewanellaceae* was abundant in our samples. This family had not been reported in previous high-throughput sequencing studies on the microbiota of African mosquitoes [19,21,22] until recent association with *Anopheles* in Burkina Faso [50]. Prior to this, its only report in *Anopheles* microbiota, to our knowledge, was an association to larvae of *An*. *stephensi* collected from coastal regions in Iran [51]. As dominating bacterial families are the most likely to be of potential use in paratransgenesis approaches, *i*.*e*. the use of midgut symbionts to interrupt parasite transmission, the identification of this new dominant bacterial family may open new perspectives in this field. Although it has been suggested that *Shewanella sp*. is associated with salty areas [51], we found *Shewanella sp*. in all our collection sites including those several kilometres inland and away from the coast. The closest collection site to the coastline was Ki_KBU (~1.5km). It is suggestive that certain conditions in all the larval environments sampled are conducive to the growth of these bacteria. However, we could only describe the observed bacteria structure based the environmental variables we used which are nominal rather than quantitative.

The abundance of salt-thriving or halophile bacteria may be as a result of the nature of activities that occur at the collection sites. The sites in Accra Metropolis are irrigation sites where vegetables and legumes are cultivated on a fairly large scale. The chemical contents of breeding sites could influence the types of bacteria that are found there and what the mosquitoes harbour in their midgut for their physiological well-being. For example, in a phosphorus-rich medium, *Pseudomonas aeruginosa* improved the larval development of *Culex quinquefasciatus* [52]. During sampling in Kintampo North, we also observed that farmers washed their tractors at some of the breeding sites we sampled, an activity that could potentially result in high chemical contents in the water. The genetic functions or metabolic potentials of *Shewanella sp*. are not known, and their predominance in our samples indicates they may play important roles in the mosquito.

One-day old, unfed *An*. *gambiae* from Kenya where shown to have predominantly *Enterobacteriaceae* in their midguts [1]. *Enterobacteriaceae* were low in abundance in most or our samples, but dominated the composition of the microbiota in a small number of samples. A similar variability in the proportion of *Enterobacteriaceae* has been previously reported in the microbiota of 13-day old mosquitoes emerged from collected larvae in Cameroon [21]. Some members of this bacterial family are known to be effective against *Plasmodium* development [16,53], while this family has also been reported to be positively associated with *P*. *falciparum* infections in mosquitoes [21]. Some *Enterobacter* and *Serratia* strains isolated from *Anopheles* mosquitoes possess anti-parasitic effects which confers some resistance to the mosquito host [16,53]. *Serratia marcescens* also affects the mosquito negatively by reducing its life span when they colonize the mosquito midgut [54]. In *An*. *stephensi*, the presence of *Klebsiella* reduced the sporogonic development of *P*. *berghei* [55]. In the rainy season, the number of breeding sites with *Enterobacteriaceae* increased, and included sites in which it had previously been absent during the dry season collection (Fig 4). Two members of this family were observed in our data; *Enterobacter sp*. and *Thorsellia anophelis*. *Thorsellia anophelis*, a dominant species in the midguts of *An*. *gambiae* found in Kenya [56]. The symbiotic relationship of *Thorsellia* with the mosquito vector has been attributed to its high tolerance for the mosquito midgut alkaline environment, and utilizing blood-meal in the midgut for growth [56,57]. The physiological impact of this species to the mosquito has, however, not been investigated. The low abundance of *Enterobacter sp*., *Serratia sp*. and absence of *Klebsiella sp*. could be an indication of high susceptibility of our mosquitoes to *Plasmodium* infection. However, the abundance of *Enterobacter* may increase in most midguts after the mosquito has had a blood-meal [1].

While getting large numbers of mosquito samples is a common concern in vector biology, the amount of variation that exists in the midgut microbiota is generally not considered. Here, we have shown that mosquito collections in the dry season have higher diversity. One may argue that this may be due to the presence of both *An*. *gambiae* and *coluzzii* in that season. Our analyses have shown that both mosquito species were similar in the average proportion of each bacterial family (*p* = 0.64), and *An*. *gambiae* presented significant variation in the microbiota composition between samples in the dry season. As this species is known to prefer temporary habitats created after rainfalls, its presence in the dry season is an indication of adaptation to permanent water pools. As a result, individuals of this sibling species could be adapting in various ways to be able to survive in these permanent environments, which may account for the significant difference in the microbiota composition among samples of this species. During the rainy season when temporary puddles of water form (a preferred environment for *An*. *gambiae* [10]), no significant variation is observed as indicated in our samples. For geographical locations that have these sibling species in sympatry, it is therefore imperative to be mindful of where and when to collect mosquitoes for infection studies, and how to interpret results. For instance, if one considers collecting more mosquitoes and so collects from a dam or irrigation sites (which usually has mosquitoes all year round), the variation in the midgut microbiota during the dry season period may greatly influence results.

This study has shown remarkable differences in the midgut bacteria species composition of *Anopheles* mosquitoes from those presented in other studies [1,3], especially in identifying *Shewanellaceae* as a dominant bacteria family. We also show that environmental factors including the season and the urban locality have a stronger impact on the midgut microbiota composition of *An*. *gambiae* and *An*. *coluzzii* than the mosquito species that harbours the bacterial consortium.

## Supporting Information

S1 Fig Average relative abundance of bacterial taxa present in test and mock samples. Taxa shown are those with at least 1% abundance in either test or mock sample.
(TIFF)

S2 Fig Correlation plots of total relative abundance of taxa detected in both mock and test samples, against amplicon concentration. (TIFF)

S3 Fig **Rarefaction curves of showing the number of bacterial species observed in dry (A) and rainy (B) seasons.** Curves represent the average for each habitat generated from 1000 sub-sampled sequences for each midgut pool analysed.
(TIFF)

S4 Fig **Boxplot comparing OTU (A) and bacterial family (B) abundances following normalization of abundance data.** Black lines indicate medians.
(TIFF)

S1 Methods Description of methods used in evaluating the presence of potential contamination in mosquito samples, and normalisation of bacterial abundance data. (PDF)

S1 Table Geographic coordinates of the studied sample sites. (XLSX)

S2 Table Multiplex Identifier (MID) assignments of samples with read counts for each sample. (XLSX)

S3 Table Number of bacterial OTUs observed in mosquitoes sampled in dry and rainy season. (XLSX)

S4 Table Relative contribution of each bacteria family to each sample from each location over the two seasonal collections. Families representing ≥1% of the microbiota are shown.
(XLSX)

S5 Table Multiple pairwise comparison test for bacteria families between breeding sites in the dry season. (XLSX)

S6 Table Two-way ANOVA comparison of bacterial genera in *An*. *gambiae* species within habitats sampled in the dry season. (XLSX)
