# Supplementary figures and images for: Seasonality and Locality Affect the Diversity of Anopheles gambiae and Anopheles coluzzii Midgut Microbiota from Ghana
Source: PLoS One. 2016 Jun 20;11(6):e0157529. doi: 10.1371/journal.pone.0157529 (PMC4913965; doi:10.1371/journal.pone.0157529)

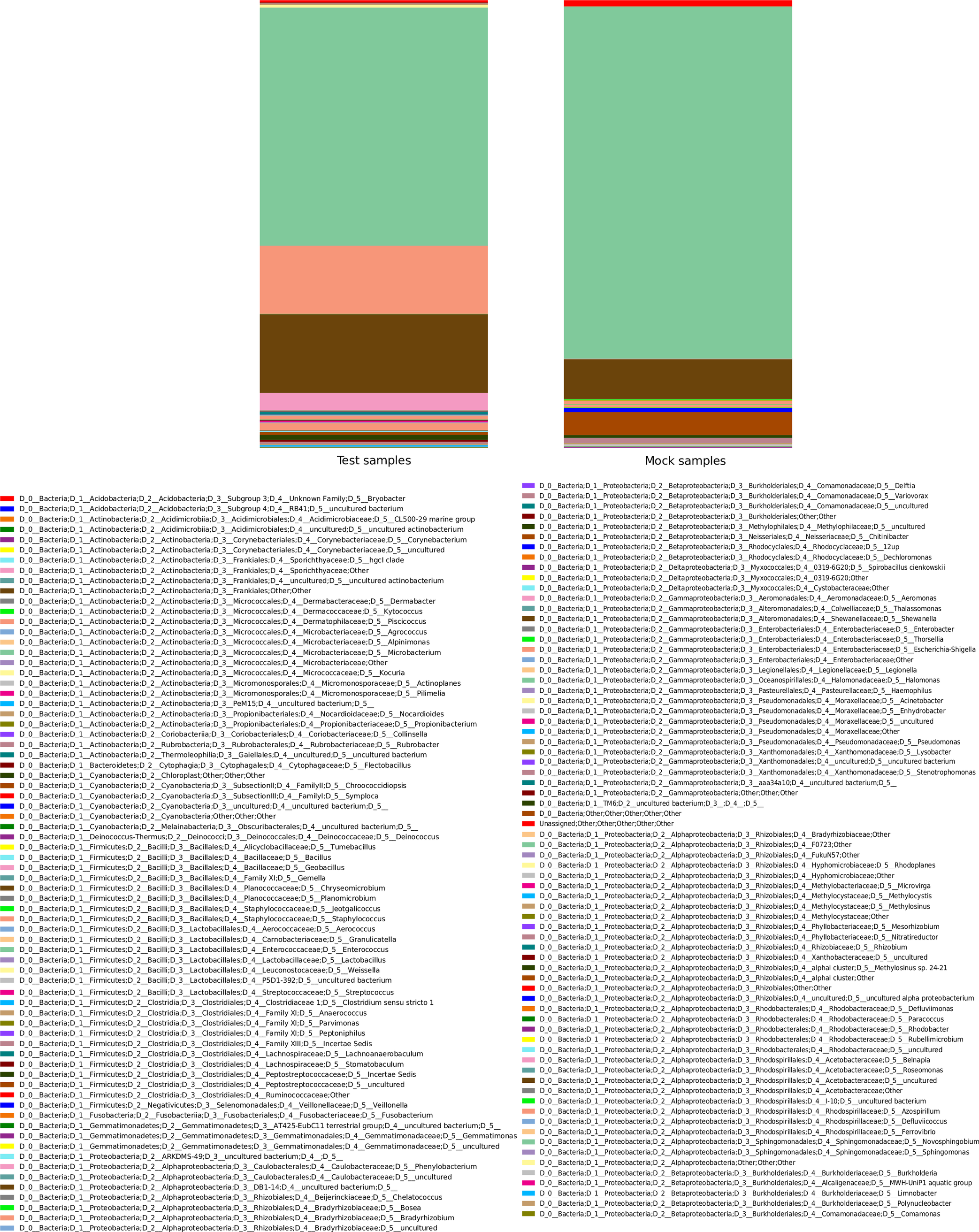

Supplement: S1 Fig — Taxa shown are those with at least 1% abundance in either test or mock sample. (TIFF) [file pone.0157529.s001.tiff]

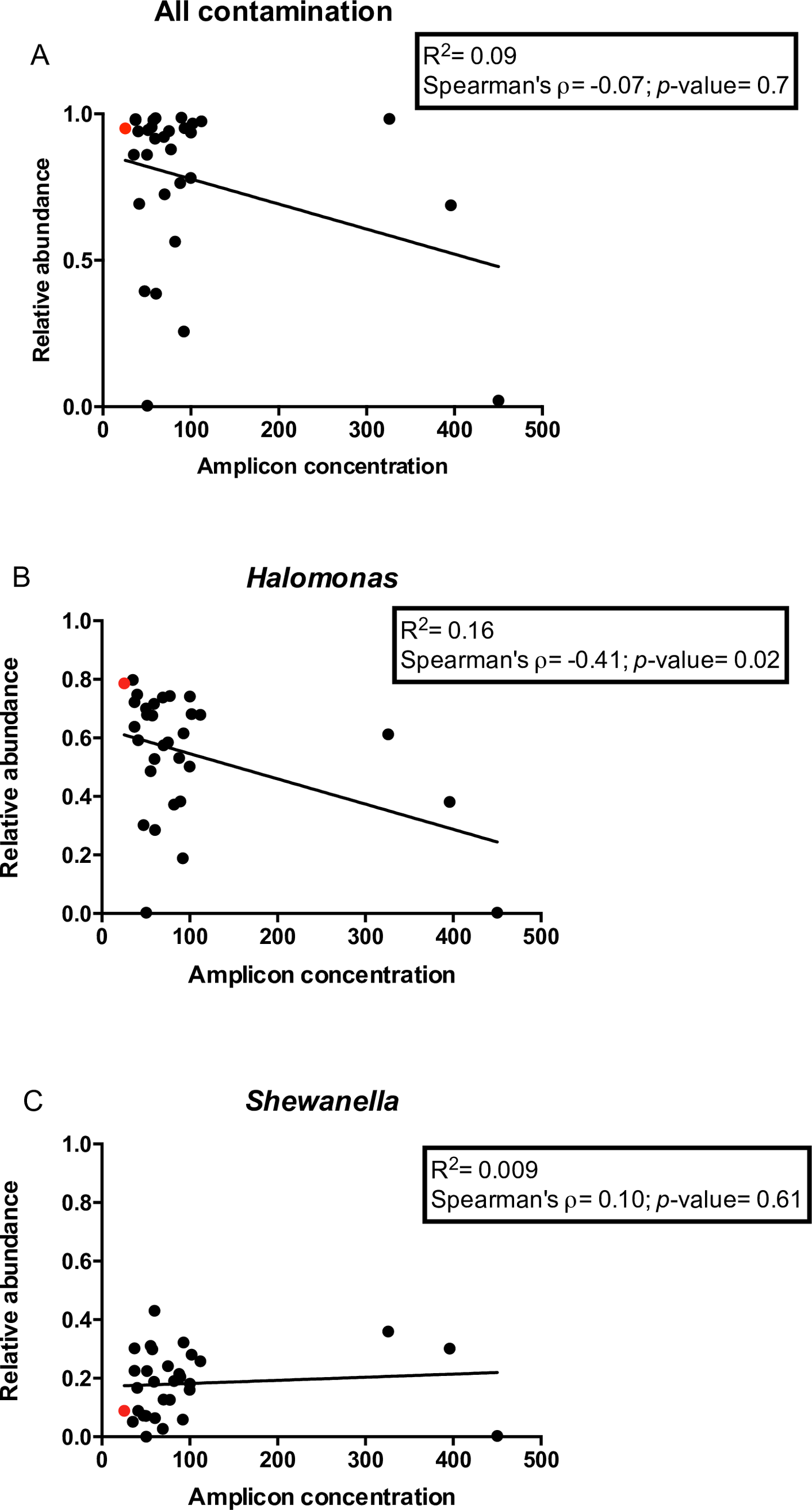

Supplement: S2 Fig — (TIFF) [file pone.0157529.s002.tiff]

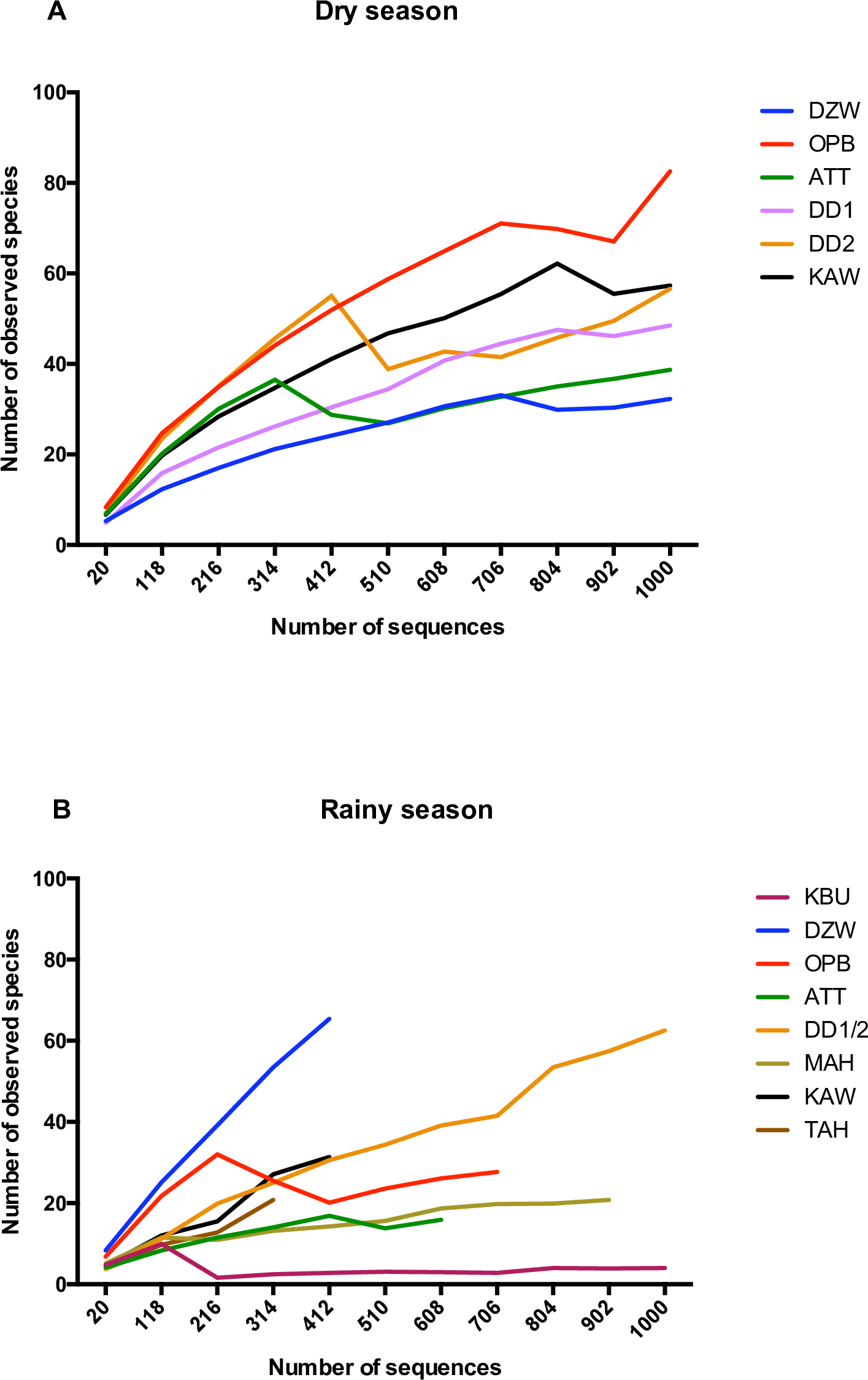

Supplement: S3 Fig — Rarefaction curves of showing the number of bacterial species observed in dry (A) and rainy (B) seasons. Curves represent the average for each habitat generated from 1000 sub-sampled sequences for each midgut pool analysed. (TIFF) [file pone.0157529.s003.tiff]

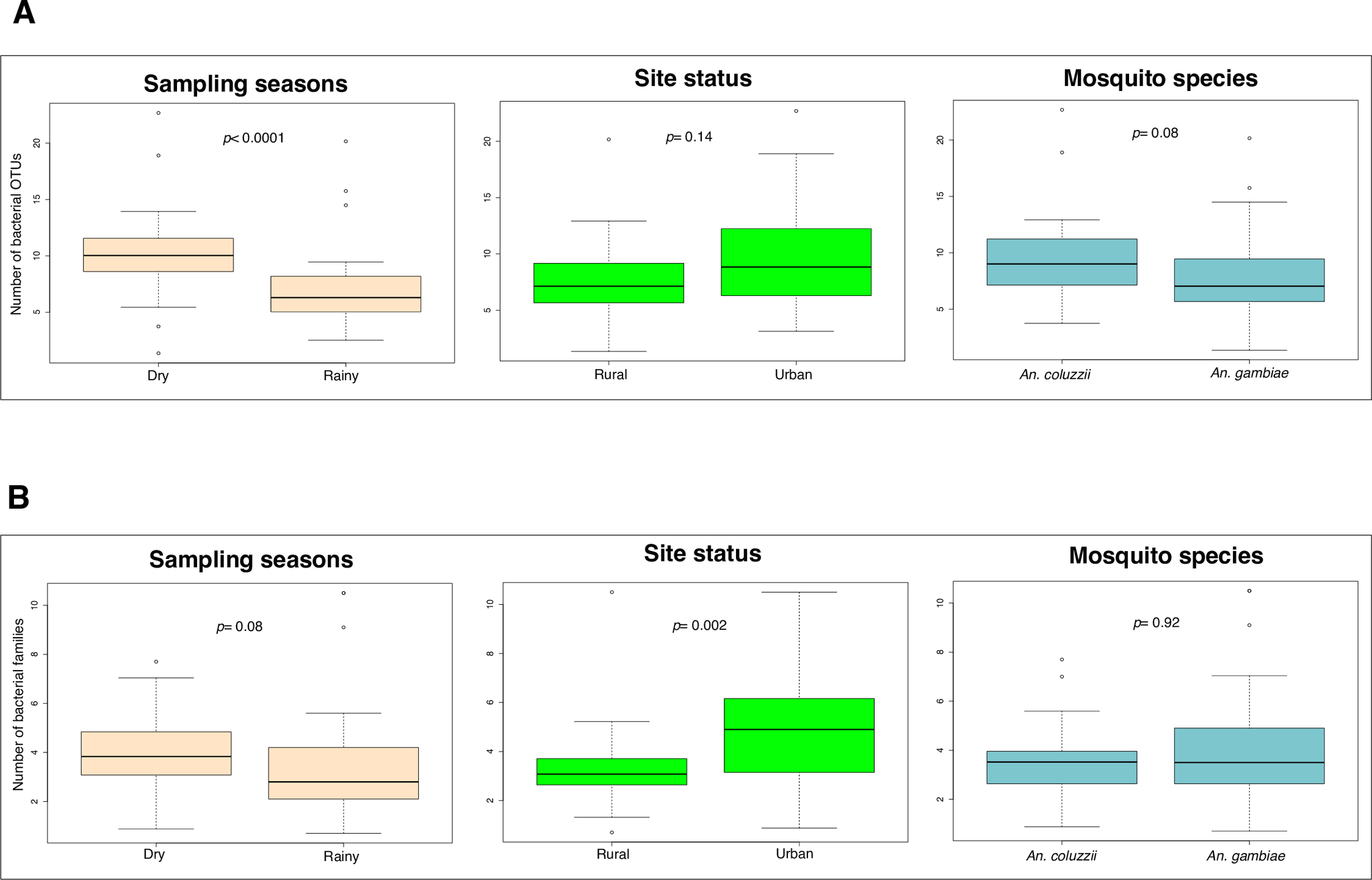

Supplement: S4 Fig — Boxplot comparing OTU (A) and bacterial family (B) abundances following normalization of abundance data. Black lines indicate medians. (TIFF) [file pone.0157529.s004.tiff]
