# Supplementary material for: Seasonality and Locality Affect the Diversity of Anopheles gambiae and Anopheles coluzzii Midgut Microbiota from Ghana
Source: PLoS One. 2016 Jun 20;11(6):e0157529. doi: 10.1371/journal.pone.0157529 (PMC4913965; doi:10.1371/journal.pone.0157529)
Supplement: S1 Methods — (PDF) [file pone.0157529.s005.pdf]

Evaluating potential contamination in test samples

To quantitate DNA in mock samples, we used 10μL of sample, as compared to 1μL of gut DNA sample. This is because, DNA may be too low in the mocks to detect in 1μL. With the Qubit fluorometer High Sensitivity (HS) DNA kit, the result always showed "<0.50ng/mL" for concentration of nucleic acids. This indicates that there is no DNA in the mock sample, and our samples were free from external contamination during dissection or extraction step. However, following PCR amplification, the mock samples produced considerable amplicon concentrations (average: 25.5ng/μL) (data not shown). Relatively high prevalence of some bacteria taxa were detected in the mock (Fig S1).

External laboratory contamination would produce the same amount of contaminants in all samples. This implies that samples with low concentration will produce comparatively high abundance of these contaminants. Thus, a plot of the relative abundance of bacteria taxa against amplicon concentrations will produce a significant negative correlation (Jervis-Bardy *et al.* 2015). To evaluate this in our dry season dataset for which the mock was sequenced, we summed the relative abundance of all taxa that were observed in both the test samples and the mock, and plotted these against the amplicon concentration, and then *Halomonas sp.* and *Shewanlla sp.* (S2 Fig). Although, we did not observe any significant correlation (p=0.7) between amplicon concentration and relative abundance when all the taxa was analysed together, a significant negative correlation was realized for the *Halomonas sp.* analyses (Spearman's rho= -0.41, *p*= 0.02). The family *Halomonadaceae* was therefore excluded from our sequences and downstream analyses.

Reference

Jervis-Bardy, J., Leong, L.E.., Marri, S., Smith, R.J., Choo, J.M., Smith-Vaughan, H.C., Nosworthy, E., Morris, P.S., O'Leary, S., Rogers, G.B., et al. (2015). Deriving accurate microbiota profiles from human samples with low bacterial content through post-sequencing processing of Illumina MiSeq data. Microbiome *3*, 1–11.

Normalization of OTU and bacterial family abundance data

We assessed whether the number of midguts per sample did not influence the differences in diversity we were observing between our variables (Fig 2). The normalization was calculated by assessing the proportion of a total amount of species found in a single gut among a group of 2-5 mosquitoes, using individual microbiota data from our previous study performed on 12 female *Anopheles gambiae* (Osei-Poku et al., 2012). We used a custom R script to obtain normalization factors based on the number of midguts in our samples, i.e 2, 3, 4 or 5. The number of OTUs found in each sample was multiplied by the following normalization factors: 0.63 for 2 mosquitoes, 0.49 for 3, 0.41 for 4 and 0.34 for 5 mosquitoes. The number of bacterial families was multiplied by 0.70, 0.58, 0.53, 0.44 for 2, 3, 4 and 5 midguts per sample, respectively.

Reference

Osei-Poku, J., Mbogo, C.M., Palmer, W.J., and Jiggins, F.M. (2012). Deep sequencing reveals extensive variation in the gut microbiota of wild mosquitoes from Kenya. Mol. Ecol. *21*, 5138–5150.
